# Statistical analysis plan for the COMPARE trial: a 3-arm randomised controlled trial comparing the effectiveness of Constraint-induced Aphasia Therapy Plus and Multi-modality Aphasia Therapy to usual care in chronic post-stroke aphasia (COMPARE)

**DOI:** 10.1186/s13063-021-05238-0

**Published:** 2021-04-23

**Authors:** Miranda L. Rose, Tapan Rai, David Copland, Lyndsey Nickels, Leanne Togher, Marcus Meinzer, Erin Godecke, Joosup Kim, Dominique A. Cadilhac, Melanie Hurley, Cassie Wilcox, Marcella Carragher

**Affiliations:** 1 School of Allied Health, Human Services and Sport, La Trobe University Melbourne, Kingsbury Drive, Bundoora, Victoria 3084 Australia; 2 Centre of Research Excellence in Aphasia Recovery and Rehabilitation, Melbourne, Australia; 3 University of Technology Sydney, Ultimo, New South Wales Australia; 4 University of Queensland, Brisbane, Queensland Australia; 5 Department of Cognitive Science, Macquarie University, Sydney, New South Wales Australia; 6 The University of Sydney, Sydney, New South Wales Australia; 7 Department of Neurology, University Medicine Greifswald, Greifswald, Germany; 8 School of Medical and Health Sciences, Edith Cowan University, Joondalup, Western Australia; 9 Australia School of Clinical Sciences at Monash Health, Monash University, Clayton, Victoria Australia; 10 Monash Health, Clayton, Victoria Australia

**Keywords:** Aphasia, Chronic, Stroke rehabilitation, Statistical analysis plan, Randomised controlled trial

## Abstract

**Background:**

While high-quality meta-analyses have confirmed the effectiveness of aphasia therapy after stroke, there is limited evidence for the comparative effectiveness of different aphasia interventions. Two commonly used interventions, Constraint-induced Aphasia Therapy Plus (CIAT Plus) and Multi-modality Aphasia Therapy (M-MAT), are hypothesised to rely on diverse underlying neural mechanisms for recovery and may be differentially responsive to aphasia severity. COMPARE is a prospective randomised open-blinded end-point trial designed to determine whether, in people with chronic post-stroke aphasia living in the community, CIAT Plus and M-MAT provide greater therapeutic benefit compared to usual care, are differentially effective according to aphasia severity, and are cost-effective. This paper details the statistical analysis plan for the COMPARE trial developed prior to data analysis.

**Methods:**

Participants (*n* = 216) are randomised to one of three arms, CIAT Plus, M-MAT or usual care, and undertake therapy with a study trained speech pathologist in groups of three participants stratified by aphasia severity. Therapy occurs for 3 h blocks per day for 10 days across 2 weeks. The primary clinical outcome is aphasia severity as measured by the Western Aphasia Battery-Revised Aphasia Quotient (WAB-R-AQ) immediately post intervention. Secondary outcomes include WAB-R-AQ at 12-week follow-up, and functional communication, discourse efficiency, multimodal communication, and health-related quality of life immediately post intervention and at 12-week follow-up.

**Results:**

Linear mixed models (LMMs) will be used to analyse differences between M-MAT and UC, and CIAT-Plus and UC on each outcome measure immediately and at 12 weeks post-intervention. The LMM for WAB-R-AQ will assess the differences in efficacy between M-MAT and CIAT-Plus. All analyses will control for baseline aphasia severity (fixed effect) and for the clustering effect of treatment groups (random effect).

**Discussion:**

This trial will provide relative effectiveness data for two common interventions for people with chronic post-stroke aphasia, and highlight possible differential effects based on aphasia severity. Together with the health economic analysis data, the results will enable more informed personalised prescription for aphasia therapy after stroke.

**Trial registration:**

Australian New Zealand Clinical Trials Registry: ACTRN 12615000618550. Registered on 15 June 2016

## Introduction

Aphasia is an acquired language disability, impacting all aspects of communication underpinned by language: speech, reading, writing, and speaking. Aphasia is present in approximately one third of stroke survivors [1], with significant negative impacts on mental health [2] and quality of life [3]. The 2016 Cochrane review of speech and language therapy for aphasia after stroke analysed 57 aphasia therapy trials and showed statistically significant treatment effects immediately after intervention but not at follow-up [4]. Although there is a broad range of approaches to aphasia intervention there is extremely limited evidence concerning their differential effects [4]. Two commonly utilised therapies, Constraint-induced Aphasia Therapy Plus (CIAT Plus) [5] and Multi-modality Aphasia Therapy (M-MAT) [6] are both intensive, high-dose interventions aimed at improving verbal communication. However, they are hypothesised to rely on different underlying neural recovery mechanisms and importantly may be differentially effective based on overall aphasia severity. Determining the most effective intervention for sub-groups of patients with aphasia may lead to improved patient outcomes and reduced health care costs.

COMPARE is a prospective, assessor-blinded, randomised clinical trial with a group- randomised design, operating in community sites across Australia and New Zealand. The primary aim of the COMPARE trial is to determine whether two contrasting, intensive treatments for chronic post-stroke aphasia, CIAT Plus and M-MAT, are superior to non-standardised, limited aphasia therapy in the community (usual care: UC). The primary endpoint is improvement on the WAB-R-AQ [7] at therapy completion. A range of other aims are addressed through secondary hypotheses listed below. Ethics and local governance approval were obtained from all hospital and university sites supporting recruitment. Version 5.2 of the protocol, dated 7 May 2019, is current and the study protocol has been published [8].

### Primary hypothesis

Compared to UC, both CIAT Plus and M-MAT will result in reduced aphasia severity as measured by the WAB-R-AQ immediately post intervention.

### Secondary hypotheses

Compared to UC, CIAT Plus and M-MAT will result in:

1. Reduced aphasia severity on the WAB-R-AQ [7] at 12-week follow-up.
2. Significantly more efficient connected speech (Content Information Units [CIUs] per minute [9]) immediately post intervention and at 12-week follow-up.
3. Significantly better ratings of functional communication (Communicative Effectiveness Index [CETI] [10]) immediately post intervention and at 12-week follow-up.
4. Significantly better multimodal communication (Scenario Test [11]) immediately post intervention and at 12-week follow-up.
5. Significantly better quality of life (Stroke and Aphasia Quality of Life Scale-39 g [SAQOL-39 g] [12]) immediately post intervention and at 12-week follow-up;

and we will also determine whether:

6. M-MAT will be more effective in reducing aphasia severity (WAB-R-AQ [7]) immediately post intervention and at the 12-week follow-up than CIAT Plus for mild and severe aphasia, whereas CIAT Plus will be more effective than M-MAT for moderate aphasia, and
7. The interventions when compared to UC are cost-effective.

The trial protocol includes additional hypotheses related to a number of tertiary outcomes including an optional sub-study. The economic evaluation for the main trial and the analysis of the tertiary outcomes will be published separately. This paper provides a detailed description of the COMPARE statistical analysis plan (SAP Version 1.1 dated September 18, 2020) and has been prepared in accordance with the published guidelines on the context of statistical analysis plans [13].

## Summary of the study protocol

Written, informed consent is obtained from each participant and their carer/significant other using hospital- and university-approved consent processes supported by aphasia friendly consent documents. Participants enrolled in the trial are allocated to treatment groups of three participants, based on their aphasia severity (as determined by the Western Aphasia Battery-Revised-Aphasia Quotient [WAB-R-AQ] [2]) and geographic location.

### Patient population

Participants include adults (18 years of age or older) living in the community who were diagnosed with any type of aphasia (WAB-R-AQ < 93.8) following a stroke at least 6 months prior to enrolment in the trial. Participants are recruited through 22 hospitals with ethical approval for the study and direct community advertising in Australia and New Zealand. Inclusion criteria and exclusion criteria are listed in the main protocol [8].

### Randomisation

Eligible participants are randomised to one of three arms: CIAT Plus, M-MAT, or UC. The intervention protocol requires therapy to be provided face-to-face, in groups of three participants. The members of a treatment group therefore need to be in the same geographic location. The trial uses a stratified group-randomisation strategy. Once enrolled in the trial, participants are allocated to treatment groups based on their aphasia severity (WAB-R-AQ mild = 93.7–62.6; moderate = 62.5–31.3; severe < 31.3). The groups are then randomised to one of three arms (M-MAT, CIAT Plus or UC) in a 1:1:1 ratio via a central allocation system using blocked randomisation within each stratum.

### Intervention

Participants randomised to the CIAT Plus and M-MAT arms of the trial attend treatment sessions 3 h a day, five times a week for 2 weeks (30 h of treatment in total). In addition, a daily home practice communication task (15 min) is given to each participant and checked for completion and logged the following day. CIAT Plus and M-MAT are provided in community settings by a qualified and study-trained speech pathologists. The UC arm is the control arm of the trial. Participants randomised to the UC arm undergo aphasia therapy in the community at the type and frequency that is available to them at the time of their recruitment and randomisation. For some participants, UC may comprise no direct intervention, while for others it may take the form of non-intense, individual, computerised or social/support group sessions (expected to be < 2 h per week). Full details of the intervention are provided in the COMPARE Trial protocol [8].

### Baseline and follow-up assessments

Pre-screening, screening and baseline evaluation procedures are described in the main trial protocol [8]. The assessed outcomes that are required for this statistical analysis include baseline, post-intervention and 12-week follow-up assessments and are detailed in the statistical plan below. Participants are assessed for their medical and stroke history. Baseline data collection includes the documentation of: demographic details including age, sex, past medical history, languages spoken, education level, handedness [14], employment, Aboriginal or Torres Strait islander status, and living arrangements; stroke type and hemisphere, first or recurrent stroke, stroke severity (mRS) [15]; apraxia of speech (Apraxia of Speech Rating Scale) [16], self-rated fatigue and distress (10-point scale), attention (Test of Everyday Attention [17]: elevator counting and visual elevator subtests), auditory verbal immediate and working memory (visual memory spans [18]), nonverbal reasoning (Raven’s coloured matrices [19]), semantic processing (Pyramids and Palm Trees [20]), and the drawing, praxis and visual orientation tasks of the Western Aphasia Battery Part 2 [7].

### Sample size considerations

The study is powered to detect a difference of 5 points on the WAB-R-AQ [7] at therapy completion. Although the minimal clinically important difference for the WAB-R-AQ [7] has not been formally determined, the standard error of measurement for participants with chronic aphasia is 4.33 points [21] with a 5-point difference considered clinically meaningful [22]. We used this information to estimate the required sample size in two steps. First, we conducted a naïve power analysis (which did not account for the clustering effect of conducting therapy in groups) which indicated a sample size of 198 to achieve 80% power at the 5% significance level. In the second step, we adjusted for the clustering effect of group therapy since the outcomes of participants from within the treatment cluster may be correlated. We anticipated a relatively small intraclass correlation coefficient (ICC) of 0.04. This is consistent with estimates of ICC used in other studies, where prior information on the size of the intraclass correlation was not available. Based on this intraclass correlation coefficient and a group size of 3, we calculated a maximum design effect of 1.08. To adjust for the clustering effect, we multiplied this value by the design effect, and rounded off to a multiple of 3, to obtain balanced allocation across the three treatment groups. This yielded the required sample size of 216.

### Other data collection

Other data collected, including tertiary outcomes, resource use (for the economic evaluation) and therapy characteristics are described in the main trial protocol [8]. Collection of data related to therapy characteristics and adverse events is summarised below.

During the trial intervention period, a daily log is completed for each trial participant which includes content, duration and frequency of sessions, nature of home tasks prescribed and completed, and participant self-rated fatigue and distress. All trial data is logged in individual electronic case report forms in a customised REDcap database. Deviation from the prescribed therapy protocol (CIAT Plus and M-MAT) is documented. Reasons for withdrawal from the trial such as health-related complications and death are recorded. A participant diary is provided to capture information on usual care therapy. Participants are questioned about general health to determine if there have been any adverse or serious adverse events during the trial period.

### Blinding

COMPARE is a complex behavioural intervention in which the treating therapists are aware of the treatment that they are providing. Participants assigned to UC will be aware that they are not receiving intense treatment. Therefore, neither the participants nor the treating therapists are considered to be blinded. However, all assessments (baseline, immediate post intervention, follow-up) are conducted by independent assessors who are blinded to treatment allocation.

### Unblinding

Only the Data and Safety Monitoring Committee (DSMC) have access to progressive data. The DSMC Chair is Professor Julie Bernhardt, University of Melbourne, Victoria, Australia. The DSMC review unblinded data in accordance with the DSMC Charter (Version 1.0, 10 November 2016).

## Statistical analysis plan

### Analysis principles and general considerations

All outcomes and analyses are prospectively characterised as primary or secondary. Differences in all endpoints between the three arms of the trial (CIAT Plus, M-MAT, UC) will be tested independently at the two-tailed 5% significance level. All estimates of treatment effects will be presented with 95% confidence intervals. No formal adjustments will be undertaken to constrain the Type I error associated with planned secondary or exploratory analyses. The information provided by analyses is designed to supplement the evidence from the primary analyses; it will provide a more complete characterisation of the treatment effects.

### Intention to treat, sensitivity and per-protocol analyses

The analyses for all outcome measures (aphasia severity, communication efficiency, functional communication, total communication, quality of life) will be conducted on an intention the treat (ITT) basis, that is, all participants will be analysed as members of the group to which they were randomised, irrespective of whether they received the allocated treatment or not. The ITT strategy for COMPARE is based on the following principles:

- All available outcome data are collected on all randomised participants
- All participants are analysed in the groups to which they are randomised.
- All available outcome data will be used in the primary analyses. The primary analyses will be reported without imputation of missing data. If the amount of missing data exceeds 10% at the primary endpoint (therapy completion), missing data will be imputed under the assumption that data is missing at random.
- A sensitivity analysis including all randomised individuals will be conducted. The sensitivity analysis will consider alternative assumptions about data missing not at random (MNAR).

Given COVID-19 pandemic impacts on face to face assessments from March 23, 2020, some trial assessments may be undertaken via videoconferencing. Assessments undertaken via videoconferencing are considered to be reliable and are expected to be consistent with face-to-face assessments [23]. These will therefore be included in the ITT analysis. The assessments conducted via videoconferencing will be assessed for consistency with the face-to-face assessments by comparing each online assessment to:

- The mean for participants with similar baseline characteristics, who received face-to-face assessments at the given timepoint.
- The mean change from previous timepoint for participants with similar baseline characteristics, who received face-to-face assessments at the given timepoint.
- A sensitivity analysis will be performed if the assessments conducted via videoconferencing (or change from previous timepoints) are found to be more than 3 standard deviations from the relevant mean face-to-face assessment (or relevant mean change).

A per-protocol analysis may be conducted separately but will not be included in the primary results publication for this trial. The per-protocol analysis will be described in a separate publication and will be based on the following principles:

- The per-protocol cohorts will be based on whether or not participants received the planned minimum 27 h/27 sessions of aphasia therapy completed in the CIAT Plus/M-MAT therapy groups.
- Participants from the CIAT Plus/M-MAT groups who do not achieve the minimum therapy amount specified in the protocol will be treated as not compliant with the treatment protocol and will be included in the control group.
- All participants who are randomised to the UC group will be included in the control group.

For primary and secondary analyses, the treatment effects for the primary effectiveness outcomes will be adjusted for baseline aphasia severity, measured by the WAB-R-AQ [7] and baseline stroke severity measured by the mRS [15]. Unadjusted analyses will be reported separately from these pre-specified analyses.

Subgroup analyses will be carried out irrespective of whether there is a significant treatment effect on the primary outcome. Their purpose is to supplement evidence from the primary analyses to help to fully characterise the treatment effect. Results from subgroup analyses will be interpreted in this context.

All analyses will be conducted using the R Statistical Programming Language [24].

### Interim analysis and stopping rules

There are no formal interim analyses planned for this trial. The DSMC periodically reviews data for the primary outcome measure and safety, and advises the chair of the management committee if, in their view, the randomised comparisons have provided both (i) ‘proof beyond reasonable doubt’ that one of the active arm interventions (CIAT Plus or M-MAT) is clearly superior or clearly inferior to usual care or because of safety concerns and (ii) evidence that might influence future patient management. The DSMC are guided by Haybittle-Peto boundaries in making this determination. That is, they work on the principle that a difference of at least 3 standard errors in the analysis of effectiveness or serious adverse events may be needed to justify halting or modifying the study before the planned recruitment is completed.

### Trial profile

**Fig. 1:**
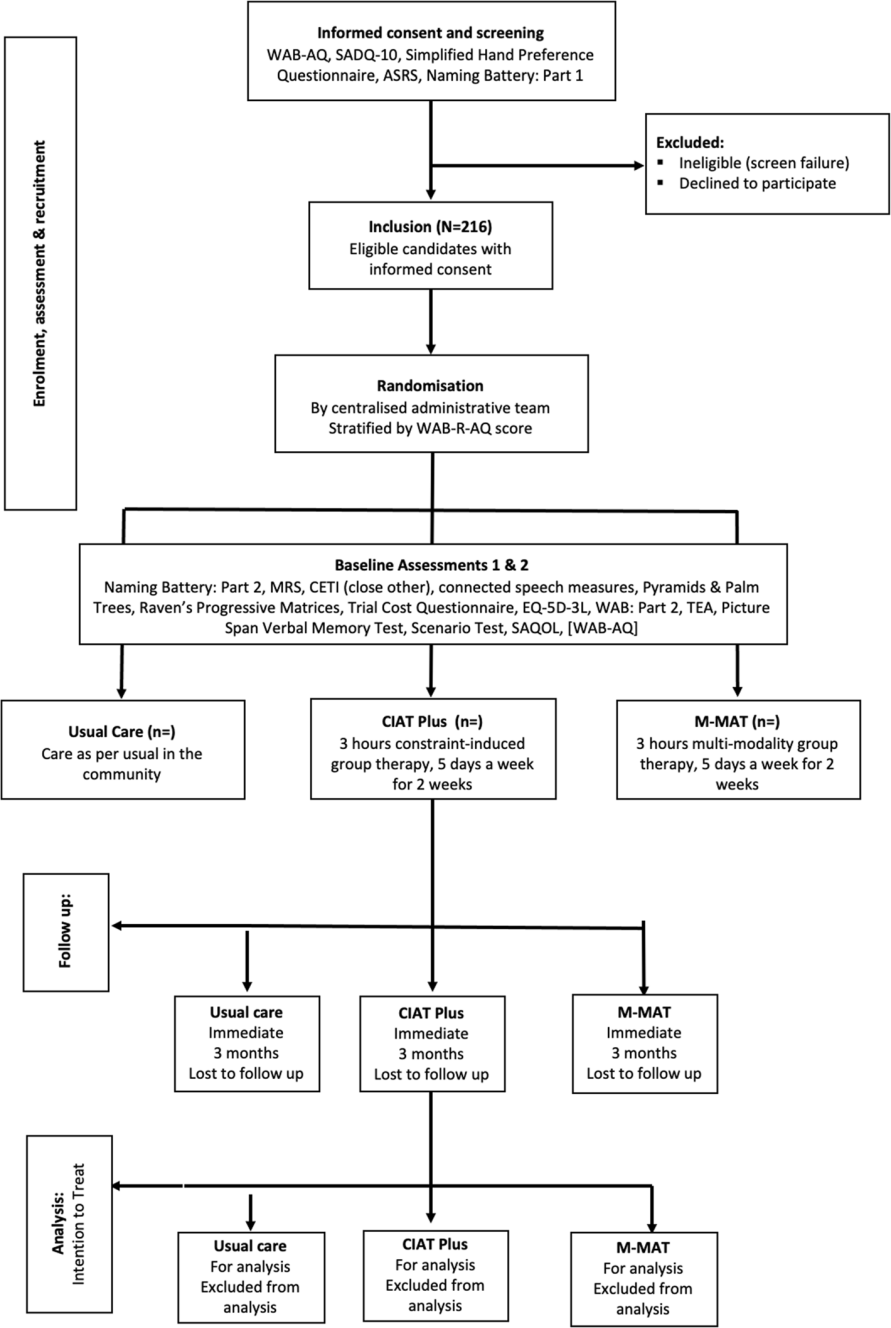
CONSORT flow diagram

The trial will be reported in accordance with the CONSORT statement for non-pharmaceutical trials and the COMPARE therapeutic protocol conforms to the SPIRIT statement. The report will include the number of screened patients who met the inclusion criteria, the number included, and the major reasons for exclusion of eligible patients. At follow-up, the number of patients withdrawn, lost to follow-up and the number who died within that period will be reported (see Fig. 1.).

### Patient characteristic and baseline comparisons

Baseline participant and stroke characteristics will be presented for each of the three groups: CIAT Plus, M-MAT and UC (Table 1). These will include age, gender, geographic region (Australia/NZ), living arrangements, stroke type, stroke severity (mRS) [15], and time since stroke. Baseline aphasia severity (WAB-R-AQ) [7], aphasia type (WAB-R-AQ) [7], apraxia of speech severity (ASRS) [16], naming (COMPARE naming battery) [8], attention (TEA) [17], memory (Visual Span) [18], non-verbal reasoning (Raven’s Coloured Matrices) [19], drawing (WAB-R Part 2) [7], praxis (WAB-R Part 2) [7], and communication efficiency (CIUs/minute) [9] will be presented in Table 2.

**Table 1:** Baseline participant and stroke characteristics

	CIAT Plus *n* (%)	M-MAT *n* (%)	UC *n* (%)	All *n* (%)
Recruitment region				
Australia				
New Zealand				
Age, median (IQR)				
< 55				
55–70				
> 70				
Gender				
Male				
Female				
Non-binary/non-disclosed				
Time post most recent stroke onset (months), Median (IQR)				
Handedness				
Right handed				
Left handed				
No preference				
Living arrangements during study				
Home alone				
Home with other				
Supported accommodation				
Baseline mRS				
Low (0–2)				
High (3–6)				
Stroke type				
Haemorrhagic				
Infarct				
Infarct and haemorrhagic				
Unknown

**Table 2:** Baseline speech, language and cognition characteristics

	CIAT Plus *n* (%)	M-MAT *n* (%)	UC *n* (%)	All *n* (%)
**Apraxia of Speech Rating Scale**				
No impairment				
Mild impairment				
Moderate impairment				
Moderate/severe impairment				
**Western Aphasia Battery-Revised-Aphasia Quotient**				
Above cut-off (93.7–100)				
Mild (62.6–93.6)				
Moderate (31.3–62.5)				
Severe (0–31.2)				
**Western Aphasia Battery-Revised Reading, Writing, Drawing, Praxis Subtests**				
Writing, mean (SD)				
Reading, mean (SD)				
Drawing, mean (SD)				
Praxis, mean (SD)				
**COMPARE Naming Battery,** mean (SD)				
**Communication accuracy and efficiency**				
No of CIUs, mean (SD)				
% CIUs per minute, mean (SD)				
**Communicative Effectiveness Index**, Mean (SD)				
**Scenario Test**, mean (SD)				
**Stroke and Aphasia Quality of Life Scale-39 g**				
Energy, mean (SD)				
Physical, mean (SD)				
Communication, mean (SD)				
Psychosocial, mean (SD)				
**Test of Everyday Attention**				
Elevator Counting, mean (SD)				
Visual Elevator, mean (SD)				
**Picture Span Memory Test**				
Pictures forward, mean (SD)				
Pictures backwards, mean (SD)				
**Raven’s Progressive Matrices**, mean (SD)				
**Self-rated Fatigue,** mean (SD)				
**Self-rated Distress,** mean (SD)				

Discrete variables will be summarised as frequencies and percentages. Unless otherwise indicated in the tables, percentages will be calculated according to the number of participants for whom data are available. If there are more than 5% missing values, the denominator will be indicated in the corresponding summary table. Continuous variables will be summarised by the mean and standard deviation (SD) or by the median and interquartile range (IQR). Durations and time intervals will be summarised by medians and IQRs.

### Primary outcome: reduction in aphasia severity immediately post intervention

#### Primary outcome measure

The primary outcome is reduction in aphasia severity immediately post intervention. Aphasia severity will be measured by the WAB-R-AQ [7]. The WAB-R-AQ [7] is a comprehensive measure incorporating production of spoken language and auditory comprehension.

#### Statistical hypothesis

The primary research hypothesis is that compared to UC, both CIAT Plus and M-MAT will result in reduced aphasia severity on the WAB-R-AQ [7] immediately post intervention. This will be tested by refuting the following null hypotheses: (a) there is no difference in aphasia severity, between the group receiving CIAT Plus and the group receiving UC immediately post intervention, and (b) there is no difference in aphasia severity, between the group receiving M-MAT and the group receiving UC immediately post intervention.

### Treatment of missing values

The primary analyses will be based on the intention to treat principle, that is, all participants will be analysed in the arm to which they were randomised. The primary analysis will be reported without imputation of missing data. If the amount of missing data warrants imputation (i.e. the number of missing values exceeds 10%), missing data imputation will be conducted under the assumption that missing values are missing at random (MAR). That is, it is assumed that the values of the missing data may reasonably be predicted from all observed data. In particular, it will be assumed that missing values of the primary outcome measure (WAB-R-AQ at intervention end) may be estimated from variables on which data has been collected (e.g., baseline aphasia severity, baseline stroke severity, age, gender, lesion size and location), and on the observed values of WAB-R-AQ. To ensure robustness of the imputation, 20 imputed data sets will be generated with a separate model being developed for each imputation. These multiple imputations will be conducted using chained equations. The pooled result of these imputed models will be reported and compared with the primary model (without imputed data). Based on monitoring by the DSMC, 6% of the data for the primary outcome measure in the COMPARE trial is missing at the time of publication of the statistical analysis plan. Sensitivity analyses that consider various other plausible assumptions about missing data will be presented.

### Analysis method

The primary aim of this trial is to compare the effects of CIAT Plus and M-MAT to usual care. This will be achieved through the primary effectiveness hypothesis which will be analysed using a linear mixed effects regression model with WAB-R-AQ as the outcome measure. Each intervention group will be compared to the UC group on the primary outcome measure (WAB-AQ immediately post intervention). The model will adjust for differences in baseline aphasia severity and baseline stroke severity by including the baseline WAB-R-AQ and the baseline mRS as covariates in the model. The effect of conducting therapy in groups will be controlled for by including the treatment group as a random effect. The treatment effect will be reported as difference in WAB-R-AQ with the corresponding 95% confidence interval.

#### Secondary hypotheses

The following secondary hypotheses will be assessed:

1. Compared to UC, CIAT Plus and M-MAT will result in a reduction in aphasia severity on the WAB-R-AQ [7] at 12-week follow-up.
2. Compared to UC, CIAT Plus and M-MAT will result in significantly more accurate and efficient connected speech (CIUs [9] per minute) immediately post intervention and at 12-week follow-up.
3. Compared to UC, CIAT Plus and M-MAT will result in significantly better functional communication (CETI [10]) immediately post intervention and at 12-week follow-up.
4. Compared to UC, CIAT Plus and M-MAT will result in significantly better multimodal communication (Scenario Test [11]) immediately post intervention and at 12-week follow-up.
5. Compared to UC, CIAT Plus and M-MAT will result in a significantly better quality of life (SAQOL-39 [12]) immediately post intervention and at 12-week follow-up.
6. M-MAT will be more effective in reducing aphasia severity (WAB-R-AQ [7]) immediately post intervention and at the 12-week follow-up than CIAT Plus for mild and severe aphasia, whereas CIAT Plus will be more effective than M-MAT for moderate aphasia.

#### Secondary statistical hypotheses

The specified set of secondary effectiveness hypotheses involve the assessment of the following statistical hypotheses:

1.1. CIAT Plus will result in greater reductions in aphasia severity than UC, as measured by the WAB-R-AQ [7], at 12 weeks post stroke.
1.2. M-MAT will result in greater reductions in aphasia severity than UC, as measured by the WAB-R-AQ [7], at 12 weeks post stroke.
2.1. CIAT Plus will result in better efficiency of connected speech than UC, measured by CIUs/min [9], immediately post intervention and at 12 weeks follow-up.
2.2. M-MAT will result in better efficiency of connected speech than UC, measured by CIUs/min [9], immediately post intervention and at 12 weeks follow-up.
3.1. CIAT Plus will result in better functional communication than UC, measured by the CETI [10], immediately post intervention and at 12 weeks follow-up.
3.2. M-MAT will result in better functional communication of connected speech than UC, measured by the CETI [10], immediately post intervention and at 12 weeks follow-up.
4.1. CIAT Plus will result in better multimodal communication than UC, measured by the Scenario Test [11], immediately post intervention and at 12 weeks follow-up.
4.2. M-MAT will result in better multimodal communication than UC, measured by the Scenario Test [11], immediately post intervention and at 12 weeks follow-up.
5.1. CIAT Plus will result in better quality of life than UC, measured by the SAQOL-39 [12], immediately post intervention and at 12 weeks follow-up.
5.2. M-MAT will result in better quality of life than UC, measured by the SAQOL-39 [12], immediately post intervention and at 12 weeks follow-up.
6.1. For participants with severe aphasia at baseline, M-MAT will result in greater reduction in aphasia severity than CIAT Plus, as measured by the WAB-R-AQ [7], immediately post intervention and at 12 weeks follow-up.
6.2. For participants with moderate aphasia at baseline, CIAT Plus will result in greater reduction in aphasia severity than M-MAT, as measured by the WAB-R-AQ [7], immediately post intervention and at 12 weeks follow-up.
6.3. For participants with mild aphasia at baseline, M-MAT will result in greater reduction in aphasia severity than CIAT Plus, as measured by the WAB-R-AQ [7], immediately post intervention and at 12 weeks follow-up.

### Analysis methods

*Comparison of CIAT Plus and M-MAT with UC immediately post intervention and 12 weeks: Aphasia Severity (WAB-AQ), Efficiency of Connected Speech (CIUs/min), Functional Communication (CETI), Multimodal Communication (Scenario Test), and Quality of Life (SAQOL-39 g).*

Longitudinal linear mixed models will be used to compare each of the CIAT Plus and M-MAT groups to the UC group on WAB-R-AQ, CIUs/min, CETI, Scenario Test and SAQOL-39 g at therapy completion and 12 weeks. The between-group differences will be assessed through a *group × time* interaction effects. The models will adjust for baseline aphasia severity by including the WAB-R-AQ at baseline as a covariate. Baseline stroke severity will be controlled for by including mRS at baseline as a fixed factor in the models. The treatment group will be included as a random effect.

### Comparison between CIAT Plus and M-MAT groups WAB-R-AQ

A longitudinal linear mixed model will be used to assess the difference between the CIAT Plus and M-MAT groups on WAB-AQ immediately post intervention and at 12 weeks follow-up. The interaction effect: *group × baseline severity* will be included in the model to assess the effects of CIAT Plus and M-MAT on participants with different baseline severity classifications. The between-group difference at each time point will be assessed through the *group× time* interaction effect. The model will adjust for baseline aphasia severity by including the WAB-AQ at baseline as a covariate. Baseline stroke severity will be controlled for by including mRS at baseline as a fixed factor in the model. The treatment group will be included as a random effect.

### Handling of missing data

Missing data for secondary analyses will be handled as described in the section on treatment of missing values.

### Other secondary outcomes: safety important medical events (IME) adverse events (AE) and serious adverse events (SAE)

Important medical events (IMEs), adverse events (AEs) and serious adverse events (SAEs) are defined in the main trial protocol. These are expected to be rare occurrences. Since the trial participants are medically stable and living in the community, aphasia therapy is not expected to have an effect on these events. Therefore, no formal hypotheses have been stated about these outcomes. All IMEs, AEs and SAEs will be reported by the therapy group. As rare events, counts of IMEs, AEs and SAEs are expected to have a Poisson or negative binomial distribution. If the data suggests that there is a between-group difference of greater than 3 standard deviations in any of these event types, then the distributions of these events will be modelled, and appropriate generalised linear mixed models will be developed to assess differences between groups.

### Sensitivity analyses

Sensitivity analysis for the primary outcome will be conducted under various assumptions about the missing data. The main analysis is planned under an assumption of missing at random; therefore, the sensitivity of the results to plausible departures from MAR will be explored as a part of an intention-to-treat analysis strategy [25–27]. The 2010 National Research Council Panel on the Handling of Missing Data in Clinical Trials [27]. recommends a transparent and easily interpretable method for conducting a sensitivity analysis. Specifically, it recommends adding a parameter (delta) to the mean response. The parameter, delta, measures the degree of departure from missing at random. We propose using this approach to conduct a sensitivity analysis that assesses sensitivity of the results to plausible departures from the MAR assumption in the COMPARE trial. If the inference about the treatment effects can be overturned by plausible values of the delta parameter, then the results of the trial will be considered equivocal.

### Tables and figures for the Main paper

**Fig. 2:**
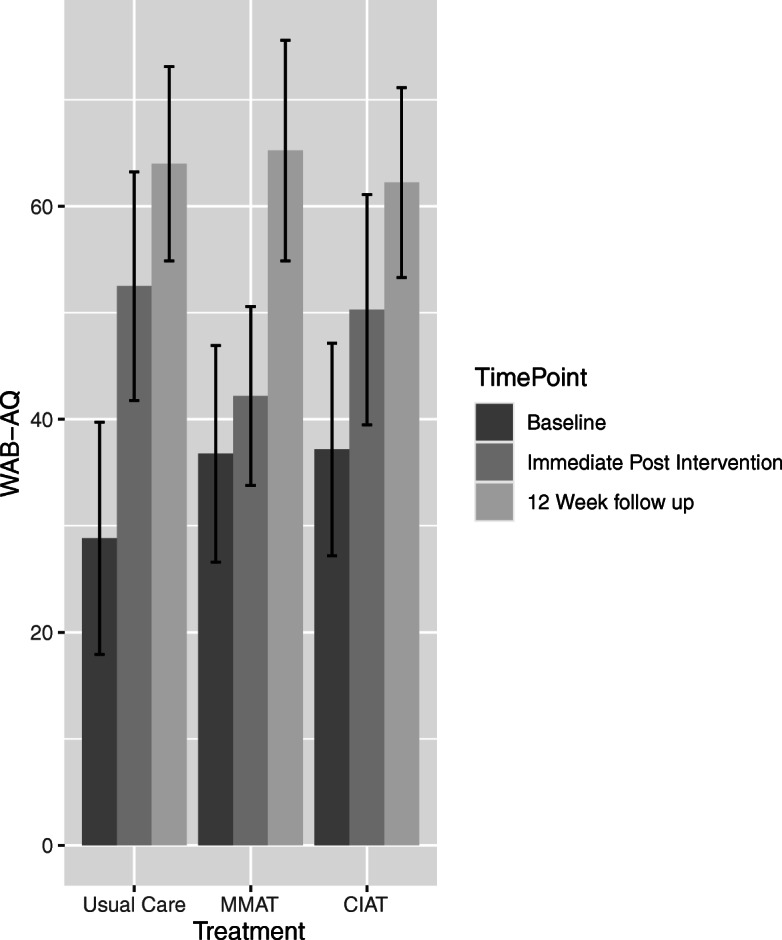
Bar plot of WAB-R-AQ at baseline, post intervention and 12-week follow-up

**Fig. 3:**
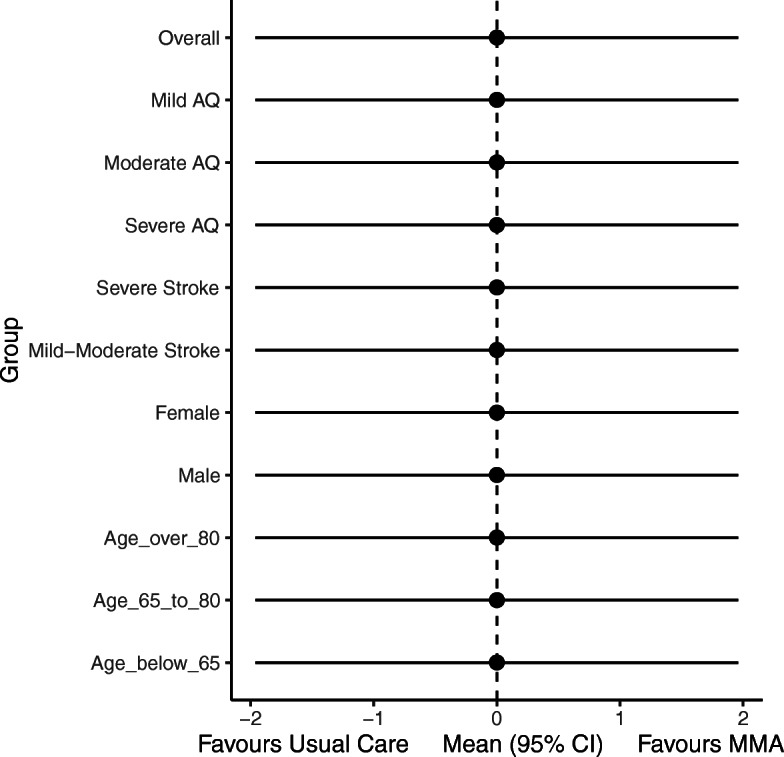
Forest plot of the treatment effect on the primary outcome for M-MAT and UC at therapy completion

**Fig. 4:**
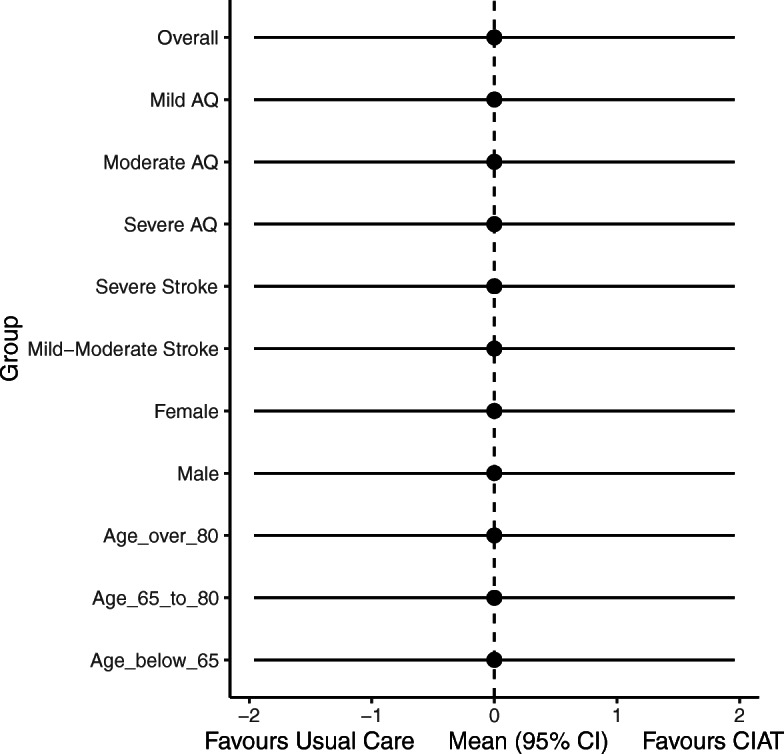
Forest plot of the treatment effect on the primary outcome for CIAT Plus and UC at therapy completion

Table 1 will report the main baseline demographic, stroke characteristics. Table 2 will report baseline speech, language, and cognition measures by participant group. Table 3 will report key summary data about the therapy characteristics (e.g. amount of therapy, number of intervention levels passed, linguistic level reached) provided for each group. Tables 4 and 5 will report the primary and main secondary outcomes at immediate post intervention and 12 weeks follow-up and Table 6 will report deaths and Serious Adverse Events. Figure 1 will be the CONSORT diagram. Figure 2 will present a bar plot of the WAB-R-AQ at baseline, post intervention and 12-week follow-up for the three arms. Figures 3 and 4 will be a forest plot of the treatment effect on the primary outcome among different subgroups at therapy completion post stroke.

**Table 3:** Intervention characteristics

	CIAT Plus *n* (%)	M-MAT *n* (%)	UC *n* (%)	All *n* (%)
Intervention compliant				
Intervention number of therapy hours, median (IQR)				
Length of sessions, mean no. minutes (SD)				
**Stimulus Set**				
Easy				
Moderate				
Hard				
**Intervention Levels progressed by final session**				
0				
1				
2				
3				
4				
5				
Number of speech therapy hours during follow-up period, median (IQR)

**Table 4:** Outcomes immediately post intervention (mean, SD)

	CIAT Plus *n*	M-MAT *n*	UC *n*	All *n*
**Primary outcome measure**				
Western Aphasia Battery-Revised-Aphasia Quotient				
**Secondary outcome measures**				
Communication accuracy and efficiency				
No of CIUs				
% CIUs per minute				
COMPARE Naming Battery				
Communicative Effectiveness Index				
Scenario Test				
Stroke and Aphasia Quality of Life Scale				
Composite Score				
Energy				
Physical				
Communication				
Psychosocial

**Table 5:** Outcomes at 12-week follow-up (mean, SD)

	CIAT Plus *n*	M-MAT *n*	UC *n*	All *n*
**Primary outcome measure**				
Western Aphasia Battery-Revised- Aphasia Quotient				
**Secondary outcome measures**				
Communication accuracy and efficiency				
No of CIUs				
% CIUs per minute				
COMPARE Naming Battery				
Communicative Effectiveness Index				
Scenario Test				
Stroke and Aphasia Quality of Life Scale				
Composite Score				
Energy				
Physical				
Communication				
Psychosocial

**Table 6:** Adverse events and serious adverse events

	CIAT Plus *n*	M-MAT *n*	UC *n*	All *n*
Adverse events				
Deaths				
Serious adverse events				
0				
1				
2				
> 2

### Summary

At the time of submission, 216 participants have been recruited and randomised in the study. The final follow-up data point was collected on July 23rd 2020, with data lock anticipated in September 2020.
